# SARS-CoV-2 Spike protein is not pro-inflammatory in human primary macrophages: endotoxin contamination and lack of protein glycosylation as possible confounders

**DOI:** 10.1007/s10565-021-09693-y

**Published:** 2022-01-11

**Authors:** Gloria Cinquegrani, Valentina Spigoni, Nicolas Thomas Iannozzi, Vanessa Parello, Riccardo C. Bonadonna, Alessandra Dei Cas

**Affiliations:** 1 Endocrinology and Metabolic Diseases, Department of Medicine and Surgery, University of Parma, Via Gramsci 14, 43126 Parma, Italy; 2 Division of Endocrinology and Metabolic Diseases, Azienda Ospedaliero-Universitaria Di Parma, Via Gramsci 14, 43126 Parma, Italy; 3 Departmental Unit of Nutritional and Metabolic Sciences, Azienda Ospedaliero-Universitaria Di Parma, Via Gramsci 14, 43126 Parma, Italy

**Keywords:** SARS-CoV-2 infection, Human macrophages, Inflammation, Lipopolysaccharide, Spike protein

## Abstract

**Introduction:**

The inflammatory potential of SARS-CoV-2 Spike S1 (Spike) has never been tested in human primary macrophages (MΦ). Different recombinant Spikes might display different effects in vitro, according to protein length and glycosylation, and endotoxin (lipopolysaccharide, LPS) contamination.

**Objectives:**

To assess (1) the effects of different Spikes on human primary MΦ inflammation; (2) whether LPS contamination of recombinant Spike is (con)cause in vitro of increased MΦ inflammation.

**Methods:**

Human primary MΦ were incubated in the presence/absence of several different Spikes (10 nM) or graded concentrations of LPS. Pro-inflammatory marker expression (qPCR and ELISA) and supernatant endotoxin contamination (LAL test) were the main readouts.

**Results:**

LPS-free, glycosylated Spike (the form expressed in infected humans) caused no inflammation in human primary MΦ. Two (out of five) Spikes were contaminated with endotoxins ≥ 3 EU/ml and triggered inflammation. A non-contaminated non-glycosylated Spike produced in *E. coli* induced MΦ inflammation.

**Conclusions:**

Glycosylated Spike per se is not pro-inflammatory for human MΦ, a feature which may be crucial to evade the host innate immunity. In vitro studies with commercially available Spike should be conducted with excruciating attention to potential LPS contamination.

**Graphical abstract:**

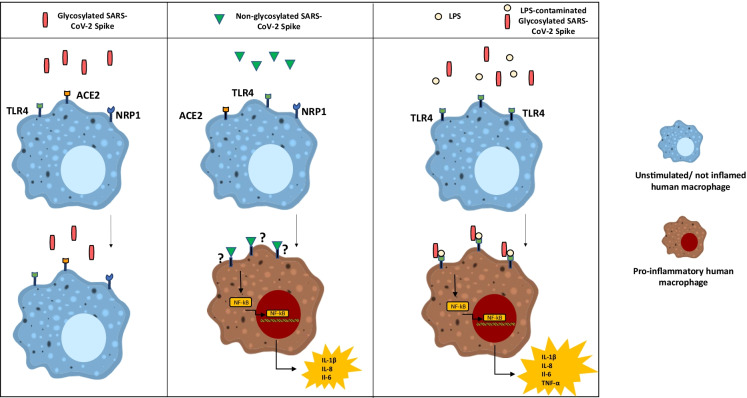

**Supplementary Information:**

The online version contains supplementary material available at 10.1007/s10565-021-09693-y.

## Introduction

Coronavirus disease 2019 (COVID-19) is caused by severe acute respiratory syndrome coronavirus 2 (SARS-CoV-2), a single-stranded RNA virus of the genus *Betacoronavirus* in the *Coronaviridae* family, which also includes SARS-CoV-1 and Middle-East Respiratory Syndrome coronavirus (MERS-CoV) (Zhu et al. 2020).

In most cases, SARS-CoV-2 engages, via the viral surface glycosylated Spike protein (Spike), the angiotensin-converting enzyme 2 (ACE2), thereby gaining access to the cytoplasm of host cells (Lan et al. 2020).

The Spike S1 domain contains the receptor-binding sequence responsible for ACE2 binding, whereas the S2 domain is crucial for cell membrane fusion (Hoffmann et al. 2020). High levels of Spike protein glycosylation are instrumental to immune escape, serving as a safety shield from the host’s innate immune system (Bagdonaite and Wandall 2018; Watanabe et al. 2020). Recent evidence demonstrated that Spike binds to additional receptors, including neuropilin-1 (NRP1) (Cantuti-Castelvetri et al. 2020; Daly et al. 2020), a multifunctional receptor for several extracellular ligands playing a crucial role in the regulation of myeloid cell function (Roy et al. 2017), and toll-like receptor (TLR)-4 (Shirato and Kizaki 2021; Choudhury and Mukherjee 2020), essential for the innate immune response to bacteria, mycobacteria, spirochetes, and viruses (Beutler 2009; Lester and Li 2014).

Macrophages (MΦ)—key cells in the innate immune response—play a pivotal role in body’s defense against viral infections mostly by producing inflammatory mediators to kill pathogens and by repairing injured tissues (Sica and Mantovani 2012; Tarique et al. 2015).

Severe COVID-19 is characterized by an aggressive inflammatory response known as cytokine storm (Ye et al. 2020), in which MΦ are implicated through an exaggerated release of inflammatory cytokines/chemokines, which fuel tissue hyperinflammation, further leukocyte recruitment, and release of inflammatory mediators, resulting into a feed-forward, pathogenic vicious cycle (Schultze and Aschenbrenner 2021). The cytokine storm, therefore, plays a major role in tissue, especially lung, damage and in the onset of the acute respiratory distress syndrome (ARDS) (Gustine and Jones 2021).

Recent studies, aimed at unveiling the molecular bases responsible for this MΦ-mediated hypercytokinemia in COVID-19, reported that Spike S1 is a key viral component in triggering MΦ inflammation, independently of virus infection or replication (Chiok et al. 2021). Specifically, SARS-CoV-2 Spike directly induces pro-inflammatory cytokine production in mouse MΦ (Shirato and Kizaki 2021; Chiok et al. 2021), MΦ derived from the human monocytic leukemia cell line THP-1 (Shirato and Kizaki 2021; Chiok et al. 2021; Pantazi et al. 2021; Khan et al. 2021), and human mononuclear cells (MNCs) (Pantazi et al. 2021), via nuclear factor-kappa B (NF-κB) activation through TLR(s) signaling. To the best of our knowledge, thus far, no studies have assessed the effects of Spike glycoprotein on inflammation in human primary MΦ.

The TLR family is associated with a strong pro-inflammatory cytokine and chemokine production—especially TLR4 which can be activated in response to LPS, derived from Gram-negative bacteria, and to few viral proteins (El-Zayat et al., 2019, Lu et al. 2008).

Accordingly, LPS is commonly used to induce in vitro MΦ polarization to classically activated M1 (Mantovani et al. 2002), which represent one extreme (alternatively activated M2 are the other extreme) of a spectrum of macrophage functional phenotypes in response to different microenvironmental cues. M1 macrophages—obtained with LPS and/or IFNγ—are responsible for killing intracellular pathogens and for releasing pro-inflammatory mediators, through TLR4 signaling cascade, which includes the recruitment of IL-1R-associated kinase 1 (IRAK-1) and the downstream activation of MAP kinases and NF-κB. These pathways eventually end up in enhancing the transcription, among others, of tumor necrosis factor alpha (TNFα), monocyte chemoattractant protein-1 (MCP1), interleukin (IL)-6, and IL-1β genes (Guzmán-Beltrán et al. 2017).

It is worth noticing that a recent study showed a specific molecular interaction between SARS-CoV-2 Spike and LPS. In that study, Spike, when combined with low levels of LPS, boosted NF-κB activation via TLR4 in monocytes, and cytokine responses in human MNCs (Petruk et al. 2020).

These findings suggest that LPS and Spike are synergistic in triggering and amplifying the inflammatory response of innate immune cells, with a potential dual relevance: (a) a subclinical Gram bacterial infection or low levels of LPS derived from gut microbiome, e.g., in the obese subject (Cani et al. 2012), may synergistically act with Spike during SARS-CoV-2 infection and significantly affect the clinical course of the disease; (b) at a less relevant level, since LPS is a quite frequent impurity of recombinant proteins derived from E. coli, but also from mammalian cells (e.g., CHO, HEK293) (Wakelin et al. 2006), undetected LPS contamination in recombinant Spike proteins might be a major confounder in experimental studies of Spike effects on innate immune cells and/or endothelium. Based on these premises, we hypothesized that LPS contamination of some commercially available recombinant SARS-CoV-2 Spike proteins might be—at least in part—responsible for the increased MΦ inflammation reported in the literature (Shirato and Kizaki 2021; Chiok et al. 2021; Pantazi et al. 2021; Khan et al. 2021).

The present investigation, therefore, was undertaken with a dual goal: (1) to investigate the in vitro effect of Spike in primary human MΦ inflammation, and, consequently to some of the evidence provided by this study, and (2) to quantify LPS contamination of some commercially available recombinant SARS-CoV-2 Spike proteins, among those reported to be pro-inflammatory in previous papers.

## Material and methods

### Cell isolation and culture

Monocyte-derived MΦ were isolated from healthy donors’ buffy coats and cultured as previously reported (Derlindati et al. 2015). Briefly, MNCs were isolated by density gradient centrifugation on Lymphoprep (Euroclone, Milano, Italy) and 2 × 10^7^ cells/ml were seeded in multiwell plates. Monocytes were selected from MNCs by plastic adherence for 1 h and cultured for 6 days in RPMI 1640 medium (Euroclone) with 10% fetal bovine serum (FBS), 1% L-glutamine, 1% pen/strep, 1% amphotericin B, and 70 ng/ml macrophage colony-stimulating factor (M-CSF) (Miltenyi Biotec, Bergisch Gladbach, Germany) at 37 °C and 5% CO_2_.

On day 6, MΦ were stimulated for 16 h with SARS-CoV-2 Spike S1 (Spike A, B, C, D, E, F purchased from the random listed companies Bio-Techne, Minneapolis, MN, USA; Arigo Biolaboratories, Hsinchu, Taiwan; Abcam, Cambridge, UK; BPS Bioscience, San Diego, CA, USA) at the concentration of 10 nM (Table 1). This concentration of 10 nM (corresponding to ~ 1 µg/ml) of Spike was selected on the basis of the literature (Shirato and Kizaki 2021; Zhao et al. 2021; Karwaciak et al. 2021).

**Table 1:** Main characteristics of the SARS-CoV-2 Spike proteins herein used

Spike protein	Amino acid sequence ^a^	Expression system	Glycosylated
Spike A	Val16-Arg685	CHO cells	Yes
Spike B	Val16-Arg685	CHO cells	Yes
Spike C	Val16-Arg685	CHO cells	Yes
Spike D	Val16-Arg685	HEK293 cells	Yes
Spike E	Met15-Cys671	*E. coli*	No
Spike F	Val16-Pro681	HEK293 cells	Yes

^a^None of the Spike proteins contains the cleavage site of furin (residues 685–686)

*Val* valine, *Arg* arginine, *Met* methionine, *Cys* cysteine, *Pro* proline, *CHO* Chinese hamster ovary, *HEK* human embryonic kidney

Where indicated, to neutralize any potential LPS interference (Tsubery et al. 2000), polymyxin B (Poly B) (2 µg/ml; Merck Life Science S.r.l., Milan, Italy) was added in culture.

To define a LPS dose–response curve, MΦ were incubated with increasing LPS concentrations (0.01—0.05—0.1—0.5—1—10—100 ng/ml; Merck Life Science) for 16 h and the effect on cell inflammation was recorded.

### Endotoxin quantification

Endotoxin contamination of recombinant spike proteins was assessed in supernatants of MΦ in the different culture conditions by Pierce Chromogenic Endotoxin Quant Kit (Thermo Fisher Scientific, Waltham, MA, USA), strictly following manufacturer’s instructions. Endotoxin concentration in diluted (from 1:10 to 1:100 depending on the degree of contamination) cell supernatants was calculated in duplicate by using the “high” standard curve provided by the kit. The reaction product was photometrically measured at 405 nm (Varioskan Lux, Thermo Scientific), and results were expressed as EU/ml.

**Fig. 1:**
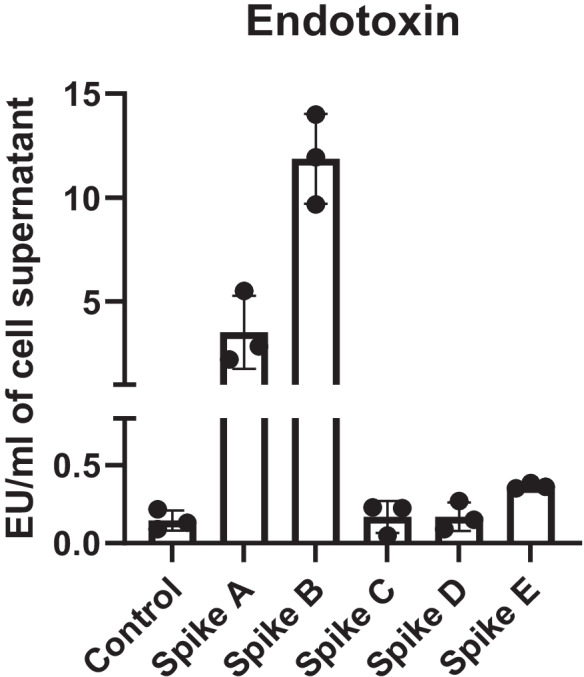
Endotoxin contamination of SARS-CoV-2 recombinant Spike proteins. Endotoxins were quantified in cell supernatants of Spike-treated MΦ. Supernatants from untreated MΦ were used as control. Data expressed as mean ± SEM from 3 independent experiments have been reported in the graph

Of note, as shown in Fig. 1, Spikes A and B were highly contaminated, while Spikes C and Spike D were uncontaminated compared to control. Spike E showed very low endotoxin level (< 0.4 EU/ml cell supernatant) (Fig. 1).

### Inflammatory marker gene expression

To quantify MΦ pro-inflammatory response, quantitative PCR (qPCR) assays were performed on candidate genes. Briefly, cells were lysed by Qiazol and total RNA was isolated by miRNeasy mini kit (Qiagen Ltd, West Sussex, UK) followed by NanoDrop (Thermo Scientific) quantification.

A total of 250 ng of RNA was retro-transcribed by using High-Capacity RNA-to-cDNA Kit (Applied Biosystem, Life Technologies, Foster City, California, USA), following manufacturer’s instructions.

Interleukin (IL)-6 (Hs00174131_m1), IL-8 (Hs00174103_m1), IL-1β (Hs01555410_m1), tumor necrosis factor (TNF)-α (Hs00174128_m1) gene expression was assessed using TaqMan Universal Master Mix (Applied Biosystems) with TaqMan primers and probes (Thermo Scientific) on a CFX Connect Real-Time (Bio-Rad, Hercules, CA, USA), as previously reported (Spigoni et al. 2020). Thermal cycling conditions were as follows: 98° for 30 s, followed by 40 amplification cycles (95 °C for 15 s; 60 °C for 1 min).

Gene expression values were calculated based on the ΔΔCt method (Schmittgen et al. 2000) and normalized to the geometric mean of RPS18 (ribosomal protein S18) (Hs01375212_g1), GAPDH (glyceraldehyde 3-phosphodehydrogenase) (Hs99999905_m1), ACTB (β-actin) (Hs99999903_m1), and B2M (β-2-Microglobulin) (Hs00187842_m1) housekeeping genes. Each sample was analyzed in triplicate and the mean values were used for calculations.

### Inflammatory molecule secretion

IL-6, IL-1β, and TNFα levels in cell culture supernatants (diluted 1:2) were quantified in duplicate by a multiparameter kit based on magnetic beads (Luminex Assay, R&D Systems) and analyzed on MagPix instrument (Luminex Corporation, Austin, TX, USA) according to kit instructions, as previously reported (Spigoni et al. 2017). IL-8 quantification was performed by Human IL-8/CXCL8 Quantikine ELISA Kit (R&D Systems) following manufacturer’s instruction. Inter- and intra-assay coefficients of variation were 6.7% and 4.6%, respectively. IL-8 concentration in diluted (1:200) cell supernatants was calculated in duplicate by using a standard curve generated by serially diluting reconstituted standards and by measuring the absorbance at 450 nm in a microplate reader (Multiskan™ FC Microplate Photometer, Thermo Scientific).

### Statistical analysis

Normally distributed data are reported as mean ± SE, while skewed data are expressed as median ± interquartile range (IQR). Differences were identified using Kruskal–Wallis with Dunn’s multiple comparison test. Paired *t-test* was performed to compare protein quantification data. Statistical significance was set at *p* < 0.05 (two-sided). Data were analyzed using GraphPad PRISM version 5.0 (GraphPad Software Inc., California, USA).

## Results

### Effects of SARS-CoV-2 Spike proteins on human MΦ inflammation

To test the effect of Spike on MΦ inflammation, we assessed pro-inflammatory marker (IL-1β, IL-6, IL-8, TNFα) gene expression in human primary MΦ treated with Spike (A, B, C, D, E) 10 nM for 16 h.

**Fig. 2:**
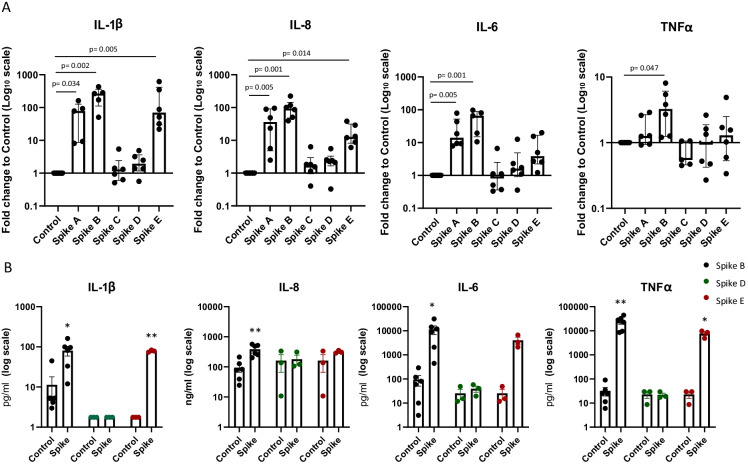
Effects of Spike proteins on pro-inflammatory cytokine/chemokine expression in human MΦ. Inflammatory marker gene (**A**) and protein (**B**) expressions were assessed in human MΦ treated/untreated with Spike (purchased from different companies) 10 nM for 16 h. Individual data points representing repeated experiments on at least 3 individual donors are shown. **A** Data are represented as median ± IQR, and differences were evaluated with Kruskal–Wallis corrected with Dunn’s multiple comparison test. **B** Data are expressed as mean ± SEM, and differences were evaluated with paired *t*-test (IL = interleukin; TNF = tumor necrosis factor) (*p < 0.05; **p < 0.01 *vs* control)

LPS-free Spikes C and D did not increase the gene expression of inflammatory biomarkers in MΦ *vs* control (Fig. 2A). Conversely—and in line with data on endotoxin contamination (Fig. 1)—Spikes A and B caused a significant increase of IL-1β, IL-8, TNFα (only Spike B), and IL-6 gene expression compared to control. Unexpectedly, Spike E—which was only slightly contaminated (0.33 EU/ml of endotoxins)—significantly boosted IL-1β and IL-8 (but not IL-6 and TNF α) gene expression compared to untreated cells (Fig. 2A).

In some selected culture conditions (Control, Spike B, D and E), cytokine release in the medium secretion was assessed to possibly confirm qPCR data (Fig. 2B). In concordance with gene expression data, LPS-free Spike D did not affect cytokine secretion *vs* control, while Spike B significantly increased the secretion of all tested biomarkers, compared to untreated control. IL-1β and TNFα secretion was also augmented by Spike E.

Thus, we inferred that Spike per se does not affect pro-inflammatory cytokine secretion in primary human MΦ.

### LPS concentration–response curve

We then performed a dose–response curve of human MΦ inflammation to scalar LPS concentrations to ascertain whether endotoxins are the only triggering factor responsible for MΦ inflammation following stimulation with LPS-contaminated Spike.

**Fig. 3:**
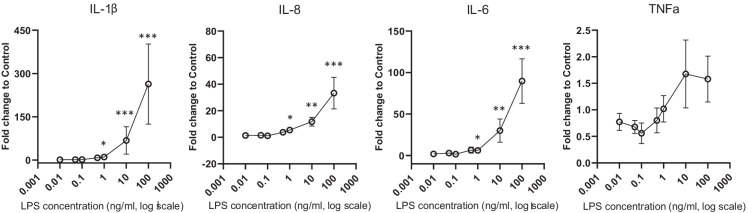
LPS dose–response curve on pro-inflammatory marker gene expression in human primary MΦ. MΦ were stimulated with increasing (0—0.01—0.05—0.1—0.5 – 1 – 10—100 ng/ml) LPS concentrations for 16 h and pro-inflammatory marker (IL-1β, IL-8, IL-6, TNFα) gene expression were assessed. Data are represented as mean ± SEM from at least 3 independent experiments and differences evaluated with Kruskal–Wallis followed by Dunn’s multiple comparison test (IL = interleukin; TNF = tumor necrosis factor) *p < 0.05; **p < 0. 005: *** *p* < 0.001 *vs* control (LPS = 0.0 ng/ml)

As shown in Fig. 3, LPS at concentrations ≥ 1 ng/ml activated MΦ, inducing an increase in pro-inflammatory marker (IL-1β, IL-6 and IL-8, but not TNFα) gene expression (Fig. 3). Lower LPS concentrations were devoid of any effect.

Furthermore, these data demonstrate that the endotoxin contamination (13 EU/ml corresponding to 1.3 ng/ml of LPS) of Spike B is sufficient to drive inflammation in cultured human MΦ.

Since LPS alone is effective only at ≥ 1 ng/ml, Spike A (showing a contamination of only 3 EU/ml of endotoxin corresponding to 0.3 ng/ml of LPS) strongly stimulates inflammation; the data are consistent with the idea that LPS and Spike are synergistic with each other, even at concentrations which are individually ineffective.

The pro-inflammatory effects associated to Spike E, however, were unexpectedly evident also at a negligible (0.03 ng/ml) LPS contamination. This prompted us to run further experiments with Spike E.

### Spike E

Spike E displayed a very low endotoxin contamination (0.3 EU/ml), but, when compared to Spikes A–D used in this study, other factors may be implicated: (i) it is shorter than the other Spikes, because it lacks the NRP1-binding domain at the C-terminal; (ii) it is produced in a prokaryotic expression system (*E. coli*), whereas Spikes A–D are expressed in mammalian cells, and all share the same amino acid sequence.

We then hypothesized that inflammation stimulated by Spike E might be attributable to:

- Spike interaction with residual (0.03 ng/ml) LPS concentrations
- The lack of the NRP1-binding domain
- The absence of post-translational modifications (glycosylation) owing to *E. coli* expression system

**Fig. 4:**
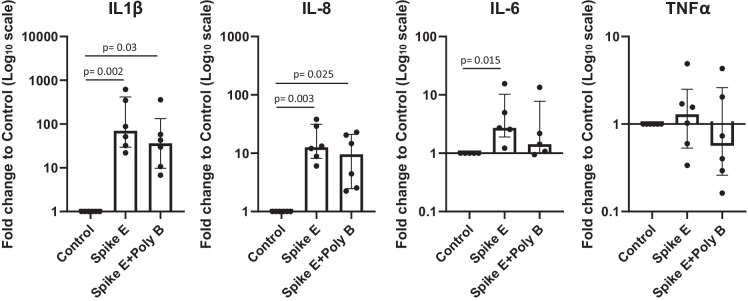
Pro-inflammatory effects of Spike E in the presence/absence of Poly B. Inflammatory marker (IL-1β, IL-8, IL-6, TNFα) gene expression was evaluated in human MΦ treated/untreated with Spike E 10 nM and Poly B 2 µg/ml for 16 h. Data are expressed as median ± IQR from at least 3 independent experiments and differences analyzed by Kruskal–Wallis followed by Dunn’s multiple comparison test (IL = interleukin; TNF = tumor necrosis factor; Poly B = polymyxin B)

To explore the potential interaction between Spike and LPS (hypothesis a), we premixed Spike E with polymyxin B—which is an antibiotic known for its capacity to bind to and neutralize LPS (Tsubery et al. 2000)—and we tested their combined effect on MΦ inflammation. As shown in Fig. 4, polymyxin B did not affect Spike E–induced inflammation, showing that its pro-inflammatory effect was not due to its minimal LPS contamination.

Next, to investigate whether the lack of NRP1-binding domain in Spike E affects inflammation (hypothesis b), we first investigated whether NRP1 was present in our cells, and we found that it is highly expressed in human MΦ (Supplementary Fig.S1).

We then tested another recombinant Spike (Spike F) which we found to be completely endotoxin-free and which also misses the NRP1-binding sequence but, at variance with Spike E, is fully glycosylated. Spike F had no effects on MΦ pro-inflammatory response compared to control (data not shown), thereby effectively ruling out hypothesis b. Hence, our data suggest that the inflammatory response induced by Spike E should be attributed to its being unglycosylated, a feature specific of the prokaryotic expression system used to produce it.

## Discussion

In the present study, we demonstrate that LPS-free recombinant glycosylated SARS-CoV-2 Spike (i.e., the Spike which is generated in infected humans) exerts no stimulatory effect on inflammation in human primary MΦ. Furthermore, we also show that the huge cytokine/chemokine production, observed in human MΦ tested with several commercially available recombinant SARS-CoV-2 Spikes, is due primarily to endotoxin (i.e., LPS) contamination of the recombinant peptides, and importantly that Spike boosts LPS-mediated pro-inflammatory action in human MΦ. We also report that a recombinant non-glycosylated Spike from *E. coli* triggers inflammation, even in the absence of endotoxin contamination, unlike glycosylated Spike produced in mammalian cells. A putative explanation of these latter findings is the lack of protein glycosylation, which is a well-known viral mechanism to elude detection by the host innate immune system (macrophages) (Watanabe et al. 2020). Thus, our results shed light on the potential confounding factors which might have affected the flourishing literature about the in vitro effects of SARS-CoV-2 Spike protein on macrophage inflammation (Shirato and Kizaki 2021; Chiok et al. 2021; Khan et al. 2021; Pantazi et al. 2021).

To the best of our knowledge, this is the first study assessing the effects of Spike glycoprotein in monocyte-derived human MΦ, and the absence of any Spike-mediated pro-inflammatory effects is in line with a very recent study showing that the treatment with Spike alone (from 0.1 to 10 µg/ml) had no effect on IL-1β secretion in MΦ from SARS-CoV-2-naïve individuals (Theobald et al. 2021).

Nevertheless, several very recent in vitro studies demonstrated a pro-inflammatory action of SARS-CoV-2 spike protein (Olajide et al. 2021; Theobald et al. 2021) in mouse (Shirato and Kizaki 2021; Khan et al. 2021) or THP-1 (Shirato and Kizaki 2021; Khan et al. 2021; Pantazi et al. 2021; Chiok et al. 2021) derived MΦ, by TLR(s) signaling activation. Importantly, none of these studies investigated a possible endotoxin contamination of the recombinant protein tested. In our work, the endotoxin contamination found in commercially available Spike casts some doubts on the significance of the aforementioned studies regarding the role of Spike in immune cell inflammation through TLR(s) activation. Indeed, in our hands, glycosylated truly LPS-free Spike has no inflammatory effects in human primary MΦ.

Our results militate against a relevant role of Spike per se in the cytokine storm of human infection played through a direct pro-inflammatory effect on MΦ. Apparently, Spike goes undetected by human MΦ only when it is glycosylated. On the other hand, the lack of any response at all to glycosylated Spike may imply that SARS-CoV-2 can evade detection by the innate immune system in the very early phases of infection (Tian et al. 2020; Kasuga et al. 2021) and this presumably is an important component of its pathogenic potential.

Endotoxin contamination of recombinant protein is a very common problem when immune cells are involved, as they can be activated by minimal amounts of LPS, equivalent to the levels of endotoxin contamination detected in some commercially available proteins (Schwarz et al. 2014). Accordingly, here we show that also human MΦ are sensitive to low (1 ng/ml) LPS concentration, which correspond to the levels of endotoxins (10 EU/ml) detected in one of the Spike tested (Spike B).

Moreover, it has been reported that recombinant peptides could be contaminated even if labelled as endotoxin-free or expressed in eukaryotic systems (Wakelin et al. 2006). We confirmed that a broad range of endotoxin contamination is found also in recombinant Spike produced in mammalian cells.

Of note, despite 1 ng/ml of LPS was identified as the concentration threshold for MΦ response, we observed that even lower endotoxin levels (i.e., 3 EU/ml) of Spike were associated to inflammation, suggesting that LPS at very low concentrations did not induce inflammation per se, but only when combined to Spike, i.e. Spike can work as a cofactor of the inflammatory action of LPS. This is in accordance with a recent paper showing that SARS-CoV-2 Spike binds to bacterial LPS, leading to changes in LPS biophysical state, thus boosting its pro-inflammatory activity in monocytes and human MNCs through TLR4-NF-κB activation (Petruk et al. 2020). The proven synergy between LPS and Spike is crucial in an attempt to explain the increased risk of severe COVID-19 in conditions characterized by subclinically increased circulating levels of LPS generated by the host gut microbiome, such as metabolic syndrome, obesity, and type 2 diabetes (Cani et al. 2012; Drucker 2021), as well as provide new therapeutic targets. In line with this observation, evidence from porcine animal models demonstrated that infection with the highly prevalent porcine respiratory coronavirus increases the lung sensibility to LPS (Van Gucht et al. 2006).We also explored the effect of a recombinant Spike (Spike E), which lacks the binding domain of NRP-1 (C-terminal peptide ^682^Arg-Arg-Ala-Arg^685^ of the S1 subunit), which is a host cell receptor reportedly able to bind Spike and to promote virus entry and infectivity (Cantuti-Castelvetri et al. 2020; Daly et al. 2020). Although our preliminary data showed a high expression of NRP-1 (and NRP-2) genes in human MΦ (Supplementary Fig. S1), we observed that the presence/absence of NRP-1-binding domain did not affect MΦ susceptibility to Spike, indicating that NRP-1 is not involved in the Spike-mediated activation of MΦ pro-inflammatory pathway.

Commercially available Spikes are produced in different expression systems, mainly *E. coli*, human HEK293, and Chinese hamster ovary (CHO) epithelial cell lines. The different expression system implies a different degree of glycosylation (null or very low in prokaryotic vs extensive in eukaryotic cells) of the final product (Dell et al. 2010; Brooks 2004). We therefore tested a Spike (Spike E) expressed in *E. coli*, with low or absent protein glycosylation, and found that it was able to elicit a robust inflammatory response in human primary MΦ.

SARS-CoV-2 Spike exhibits both N and O glycosylations (Shajahan et al. 2020; Reis et al., 2021). In particular, two N-glycosylation sites—specifically glycosylated by the machinery of the host (mammalian) cells (Kornfeld and Kornfeld 1985)—have been identified in the receptor-binding domain and are recognized as important mediators of SARS-CoV-2 binding to host cells via the ACE2 receptor (Reis et al., 2021). The different degree of glycosylation may explain the increased inflammatory profile induced by Spike E *vs* Spike F, which share the same amino acid sequence but are produced in different protein expression system (*E. coli* vs HEK293, respectively).

Of note, the presence of glycans—arranging in a shield around the RBD—is a common strategy to escape immune surveillance for coronaviruses and other viruses with heavy glycosylated spike proteins (like HIV-1 Env) (Reis et al., 2021). In this framework, our data strongly suggest that prokaryotic-expressed recombinant Spike, naturally lacking post-translational modification at specific glycosylation sites, can be recognized by host immune cells, in primis MΦ, which build a first-line antiviral response.

In line with our hypothesis, some recent works reported a pro-inflammatory effect of prokaryotic-expressed Spike proteins in immune cells (Shirato and Kizaki 2021; Rotoli et al. 2021).

Thus, speculations based on in vitro studies of immune effects of non-glycosylated Spike should be avoided, as it does not correspond to that found in virions, which is highly glycosylated.

Our studies were limited to MΦ; hence, we cannot rule out that the behavior of non-immune cells, like epithelial and endothelial cells, which also are very sensitive to LPS stimulation (Menden et al. 2013), is different. Future experiments are needed to clarify this crucial issue.

Some study limitations must be acknowledged: (1) it is uncertain whether the concentration of Spike used in the present work (10 nM) is comparable to those occurring in infected subjects—of note, the viral load of an infected individual varies from 10^2^ to 10^13^ copies/ml (Costa et al. 2021) and that the trimeric spike copy number per virion is 26 ± 15 (Yao et al. 2020): the highest figures should generate in vivo a Spike concentration around 2–7 nM; (2) although our data are compatible with a role of Spike glycosylation in macrophage inflammation, future ad hoc experiments should be performed to prove a causal relationship; (3) the extrapolation of our in vitro data to the complex in vivo setting should be made with caution.

## Conclusions

In conclusion, this study demonstrates that (a) LPS-free, glycosylated SARS-CoV-2 Spike proteins do not cause inflammation but, rather, Spike protein advances LPS-mediated pro-inflammatory action in primary human MΦ; and (b) the in vitro high cytokine release, induced by recombinant Spike in human MΦ, has to be attributed primarily to LPS contamination of the recombinant peptides. Moreover, we observed that non-glycosylated Spike—which does not represent the protein expressed on SARS-CoV-2 virions in infected mammals—is pro-inflammatory, thereby highlighting the potential role of glycosylation in SARS-CoV-2 pathogenicity.

In vitro studies with commercially available Spike should be conducted with excruciating attention to potential LPS contamination.

## Supplementary Information

Below is the link to the electronic supplementary material.

Supplementary Fig. S1. Gene expression of Spike receptors on human macrophages. Gene expression of ACE2, TMPRSS2, NRP1 and NRP2 was tested in cultured macrophages and in A549 (adenocarcinomic human alveolar basal epithelial cells) and Calu-3 (human lung cancer cell line). Gene expression data, normalized to the RPL15 (Ribosomal like protein 15) housekeeping gene, are represented as mean ± SEM from at least 3 independent experiments (PDF 86 KB)

## Data Availability

The datasets generated during and/or analyzed during the current study are available from the corresponding author on reasonable request.
