# Supplementary figures and images for: SARS-CoV-2 Spike protein is not pro-inflammatory in human primary macrophages: endotoxin contamination and lack of protein glycosylation as possible confounders
Source: Cell Biol Toxicol. 2022 Jan 11;38(4):667–78. doi: 10.1007/s10565-021-09693-y (PMC8749924; doi:10.1007/s10565-021-09693-y)

Figure S1

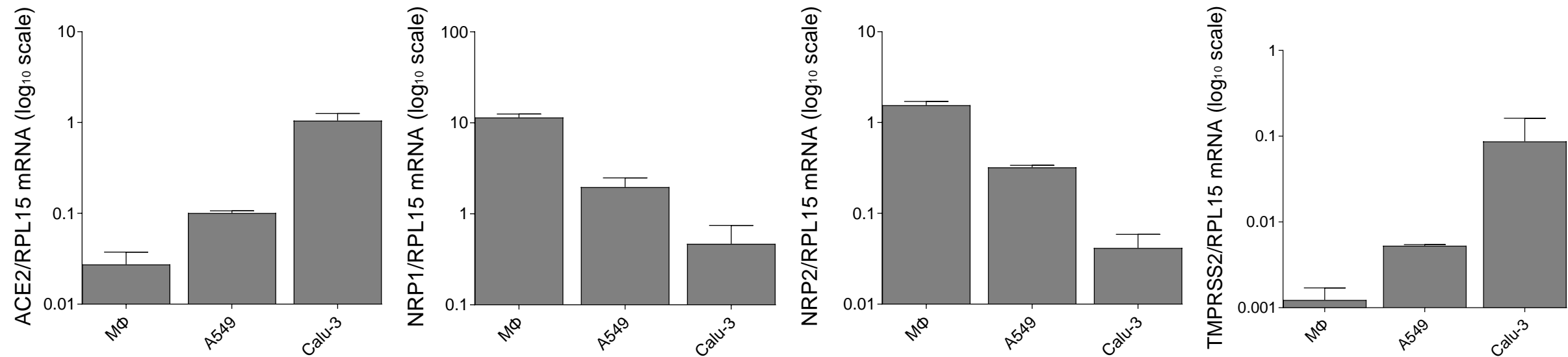

Supplement: Supplementary file 1 — Supplementary Fig. S1. Gene expression of Spike receptors on human macrophages. Gene expression of ACE2, TMPRSS2, NRP1 and NRP2 was tested in cultured macrophages and in A549 (adenocarcinomic human alveolar basal epithelial cells) and Calu-3 (human lung cancer cell line). Gene expression data, normalized to the RPL15 (Ribosomal like protein 15) housekeeping gene, are represented as mean ± SEM from at least 3 independent experiments (PDF 86 KB) [file 10565_2021_9693_MOESM1_ESM.pdf]
